# Standardized unfold mapping: a technique to permit left atrial regional data display and analysis

**DOI:** 10.1007/s10840-017-0281-3

**Published:** 2017-09-07

**Authors:** Steven E. Williams, Catalina Tobon-Gomez, Maria A. Zuluaga, Henry Chubb, Constantine Butakoff, Rashed Karim, Elena Ahmed, Oscar Camara, Kawal S. Rhode

**Affiliations:** 1 Division of Imaging Sciences and Biomedical Engineering, King’s College London, 4th Floor North Wing, St. Thomas’ Hospital, 249 Westminster Bridge Road, London, SE1 7EH UK; 2 Translational Imaging Group, Centre for Medical Image Computing, University College London, London, UK; 3 PhySense, Universitat Pompeu Fabra, Barcelona, Spain

**Keywords:** Atrial fibrillation, Catheter ablation, Cardiac magnetic resonance, Unfold map, Regional analysis, Contact-force analysis

## Abstract

**Purpose:**

Left atrial arrhythmia substrate assessment can involve multiple imaging and electrical modalities, but visual analysis of data on 3D surfaces is time-consuming and suffers from limited reproducibility. Unfold maps (e.g., the left ventricular bull’s eye plot) allow 2D visualization, facilitate multimodal data representation, and provide a common reference space for inter-subject comparison. The aim of this work is to develop a method for automatic representation of multimodal information on a left atrial standardized unfold map (LA-SUM).

**Methods:**

The LA-SUM technique was developed and validated using 18 electroanatomic mapping (EAM) LA geometries before being applied to ten cardiac magnetic resonance/EAM paired geometries. The LA-SUM was defined as an unfold template of an average LA mesh, and registration of clinical data to this mesh facilitated creation of new LA-SUMs by surface parameterization.

**Results:**

The LA-SUM represents 24 LA regions on a flattened surface. Intra-observer variability of LA-SUMs for both EAM and CMR datasets was minimal; root-mean square difference of 0.008 ± 0.010 and 0.007 ± 0.005 ms (local activation time maps), 0.068 ± 0.063 gs (force-time integral maps), and 0.031 ± 0.026 (CMR LGE signal intensity maps). Following validation, LA-SUMs were used for automatic quantification of post-ablation scar formation using CMR imaging, demonstrating a weak but significant relationship between ablation force-time integral and scar coverage (*R* ^2^ = 0.18, *P* < 0.0001).

**Conclusions:**

The proposed LA-SUM displays an integrated unfold map for multimodal information. The method is applicable to any LA surface, including those derived from imaging and EAM systems. The LA-SUM would facilitate standardization of future research studies involving segmental analysis of the LA.

**Electronic supplementary material:**

The online version of this article (10.1007/s10840-017-0281-3) contains supplementary material, which is available to authorized users.

## Introduction

Left ventricular imaging data has long been visualized and compared using surface flattening techniques (‘polar’ or ‘bull’s eye’ plots). A wide variety of data input sources have been used to create such plots, including echo strain mapping, SPECT imaging, and cardiac magnetic resonance imaging [1, 2]. By resolving different data sources onto a single spatial domain, direct comparisons between substrate analysis techniques for the left ventricle become possible [3]. Meanwhile, left atrial (LA) electrophysiological substrate assessment has progressed in recent years with voltage mapping, activation mapping, rotor mapping, complex signal analysis, CT imaging, and CMR imaging [4], all providing valuable information in various settings. To deal with such an abundance of information, we recently proposed a technique to allow simultaneous representation of multiple parameters on a single 3D cardiac chamber model [5].

Against this background, surface flattening techniques offer some advantages over 3D shells by (1) enabling simultaneous visualization of all available information and (2) facilitating intra- and inter-subject comparisons of datasets. For example, unfold maps have been used to compare pre- and post-ablation maps of the same subject [6]. Since the shape of these unfold maps changes with the geometry of the left atrium, this approach is limited to intra- rather than inter-patient comparisons. Therefore, in this study, we generate a standardized regional unfold map (LA-SUM) that follows the same template for all subjects. Akin to the ventricular ‘bull’s eye’ plot, the proposed LA-SUM allows for intra-patient comparison of multimodal data and provides a common reference space for inter-subject analysis. After developing the LA-SUM, we demonstrate its application in representing post-ablation atrial enhancement on CMR imaging, and comparing ablation parameters between cases undergoing pulmonary vein isolation.

## Methods

### Study population and data collection

**Fig. 1:**
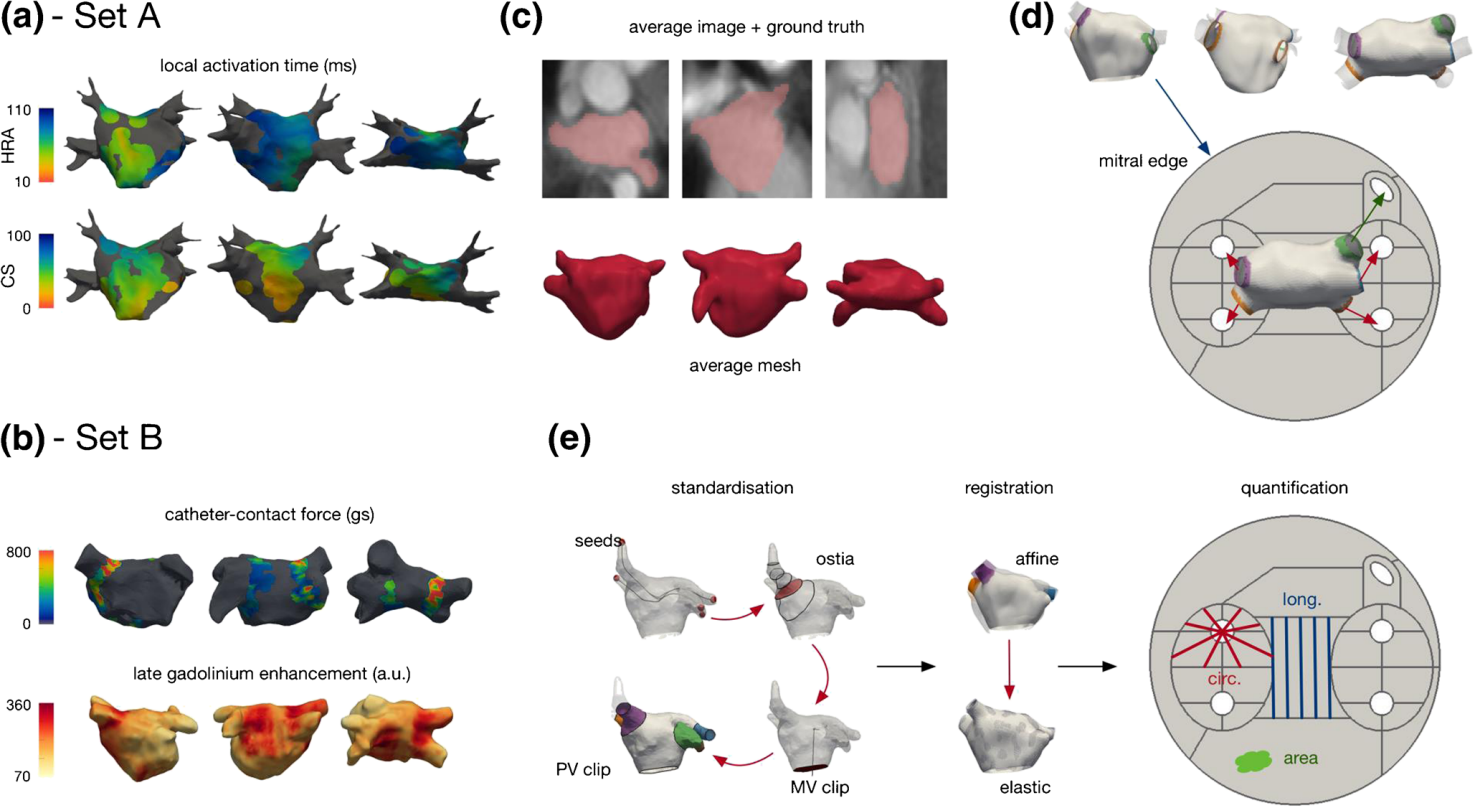
Input datasets and LA-SUM processing schematic. **a**, **b** Example datasets from set A and set B showing local activation time measurements from the coronary sinus (CS) and high right atrial (HRA), contact-force measurements during ablation and post-ablation late gadolinium enhancement. **c–e** The LA-SUM processing pipeline. An average mesh was constructed from an average atlas image (**c**). The average mesh was standardized and unfolded to fit a predefined template. The LA-SUM transforms the mitral edge to an outer circle, each PV to a spaced circle and the LAA to an ellipse (**d**). A case is processed by manually selecting four seed points (one per main PV) for mesh standardization, mitral valve clipping, and pulmonary vein clipping before the case data is projected onto the average mesh using affine followed by elastic registration. Finally, circumferential, longitudinal, and area coverage calculation is performed for quantification (see text)

The study population comprised 28 patients who underwent clinically indicated electroanatomic mapping procedures. Data collection was approved by the National Research Ethics Service (REC reference numbers: 10/H0802/77 and 08/H0802/68), and all patients gave written informed consent. The cases were divided into two sets (see Fig. 1a, b) as follows:

- *Set A* was used to develop the LA-SUM technique, including the determination of 2D LA-SUM correlates of 3D LA regions. Set A consisted of 18 patients (61 ± 12 years, 9 males, 13 paroxysmal AF, 3 persistent AF, 2 left-sided accessory pathways) undergoing left atrial local activation time (LAT) mapping prior to pulmonary vein isolation or accessory pathway ablation.
- *Set B* was used to test the application of the LA-SUM technique, including intra-patient comparison of post-ablation scar formation. Set B consisted of ten patients (60 ± 11 years, seven males, five persistent AF) undergoing wide area circumferential ablation (WACA) for pulmonary vein isolation (PVI) using a contact force sensing catheter (SmartTouch, Biosense Webster, Diamond Bar, CA, USA).

#### Electroanatomic mapping

Following trans-septal puncture, a 3D geometry of the left atrium was created using either the Velocity (St Jude Medical Inc., St Paul, MN, USA) or Carto3 (Biosense Webster, Diamond Bar, CA, USA) EAM platforms. Bipolar electrograms were collected throughout the left atrium using the PentaRay mapping catheter (Biosense Webster, Diamond Bar, CA, USA) during pacing from the high right atrium (HRA) or mid-coronary sinus (CS). Electrode positions for each LAT sampling site were recorded on the 3D geometry. LAT for each recording site was defined as the time from pacing stimulus to the earliest sharp deflection on each local electrogram. LAT maps were created by interpolating LAT times across each 3D geometry, with a distance fill threshold of 10 mm.

#### Cardiac magnetic resonance imaging

Post-ablation atrial CMR imaging was performed in set B at a median of 4 (range 3–12) months post-ablation using an Achieva 3.0T MRI scanner (Philips, Best, The Netherlands). 3D whole heart (WH) and 3D late gadolinium enhancement (LGE) datasets were obtained and processed using a maximum intensity projection in order to quantify the extent of post-ablation injury, as previously described [7] (see online supplement).

### LA-SUM generation

We defined the LA-SUM as an unfold template of an average LA mesh (Fig. 1c). The average mesh was unfolded using a fast surface parameterization technique developed for texture mapping [8]. With this mapping method, a predefined unfold template was constructed where the mitral valve annulus is constrained to a disk, the boundary of each PV to circles, and the LA appendage to an ellipse (see Fig. 1d). Circles in the unfold template corresponding to the PVs were slightly offset to preserve the proportions of the 3D average mesh (i.e., longer distance to the mitral valve annulus along the septal wall than along the lateral wall). To generate an LA-SUM, a surface mesh (e.g., from EAM or CMR imaging) is first semi-automatically pre-processed to define the mitral valve annulus [9] and the pulmonary veins are clipped 10 mm distal to the ostia. Next, the surface mesh is registered to the LA average mesh before unfolding onto the 2D LA-SUM. Further details are given in the online supplement. For each case, the LA-SUM was completed by two independent observers to evaluate the robustness of the technique to variations in the pre-processing steps. Root mean square deviation (RMSD) is reported as a measure of inter-observer variability.

**Fig. 2:**
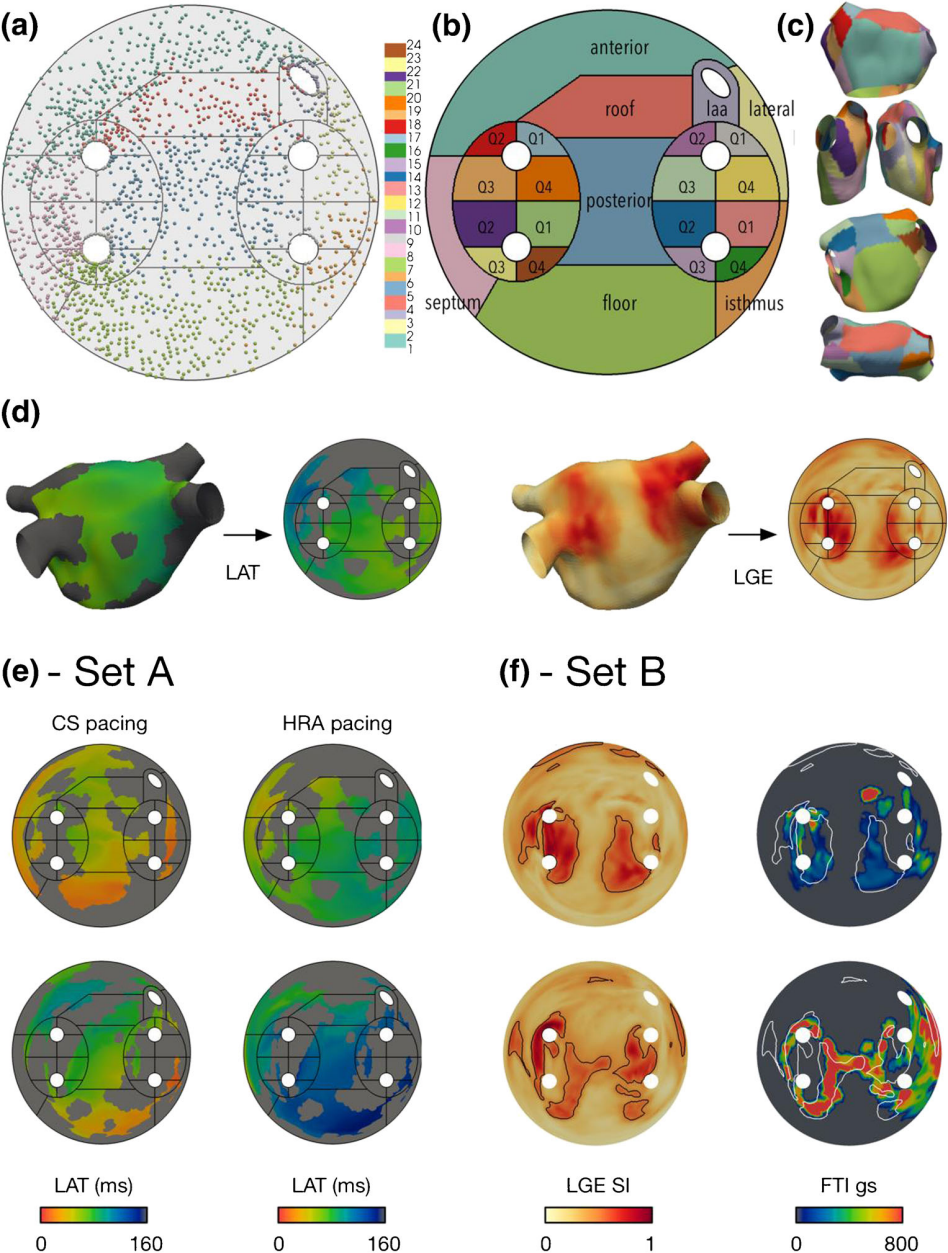
LA-SUM region definitions, representative examples and qualitative data analysis. **a**–**c** Region definitions. Region labels were marked on 3D geometries from all cases in set A and projected onto a cumulative LA-SUM to define the regions in 2D (**a**). Individual points in **a** represent contact mapping points recorded with the PentaRay catheter in all set A patients, mapped onto the SUM template. Eight common LA regions were defined (**b**), numbered as follows: region 1 = anterior—from roof to mitral annulus; region 2 = lateral—between appendage, mitral annulus, and LSPV; region 3 = LAA—left atrial appendage; region 4 = roof—posterior to RSPV-RA line and anterior to RSPV-LSPV line; region 5 = posterior—bounded by pulmonary veins; region 6 = mitral isthmus—between LSPV, mitral annulus, and LIPV; region 7 = floor—from posterior to mitral annulus; region 8 = septum—medial border of chamber between anterior and floor regions. In addition, four quadrants around each PV were defined, numbered as follows: regions 9–12 = LSPV quadrants Q1–Q4; regions 13–16 = LIPV quadrants Q1–Q4; regions 17–20 = RSPV quadrants Q1–Q4; regions 21–24 = RIPV quadrants Q1–Q4. The region definitions were mapped back to the 3D average mesh to test for consistency (**c**). **d** Example LA-SUM representations for a LAT map from set A and a post-ablation LGE CMR scan from set B. **e, f** Intra-patient comparison of LA datasets is demonstrated for two cases from set A (LAT during CS pacing and LAT during HRA pacing) and two cases from set B (force-time integral during ablation and post-ablation scar demonstrated by LGE CMR)

To split the LA-SUM into clinically relevant regions, eight LA regions (anterior, lateral, appendage, roof, posterior, mitral isthmus, floor, and septum) were defined [10]. These regions were marked on all 3D meshes from set A. An LA-SUM was computed for each case, and a cumulative LA-SUM (all patients, all regions) was visualized to define the regions in 2D (Fig. 2a). The boundaries between LA regions on the LA-SUM maps were optimized by consensus of two electrophysiologists. The final regions were mapped back to the 3D average mesh to test for consistency (Fig. 2c). Four quadrants (Q1, Q2, Q3, and Q4) were also defined around each PV. These quadrants were included in the SUM for the purpose of computing the circumferential coverage of post-ablation injury (see LA-SUM Applications, below). In total, there are 24 regions in the proposed LA-SUM.

### LA-SUM applications

#### Lesion and contact force correlation

Using the defined SUM template, the relationship between LGE and FTI was analyzed for cases in set B. To define areas of enhancement from the LGE meshes, the intensity histogram was normalized to a 16-bit window [11] from the intensity values obtained from the LGE images. Subsequently, an optimal threshold value was chosen per case [12] (50 ± 10% of maximum intensity) and contours corresponding to the extent of enhancement were overlaid onto the matching FTI LA-SUM. For each LA-SUM region, the ablation coverage (FTI > 100 gs) was correlated with the percentage of lesion coverage (see below).

#### Percentage of lesion coverage

To quantify the extent of post-ablation lesion coverage (defined by LGE CMR or EAM FTI), the LA-SUM was used to compute three metrics: *circumferential* coverage, *longitudinal* coverage, and *area* coverage (see Fig. 1e—quantification). For circumferential coverage, the four quadrants corresponding to each PV were extracted and sampled radially around the PV opening at one degree intervals. Circumferential coverage was defined as the ratio of the number of sampling lines crossing any lesion to the total number of sampling lines (*n* = 360). Longitudinal coverage was evaluated on the roof and the posterior walls by sampling 100 vertical lines along a horizontal axis of each region. Longitudinal coverage was defined as the ratio of the number of sampling lines crossing any lesion to the total number of sampling lines (*n* = 100). Area coverage was computed for all SUM regions and was defined as the ratio of the area covered by a lesion to the total region area.

## Results

Figure 2a–c shows the LA-SUM template generated using set A. Electrogram recording sites were ‘tagged’ with one of eight regions on the native 3D shell before representing all set A cases on a single LA-SUM (Fig. 2a). In doing so, LA-SUM regions were defined using the boundaries between recording sites to represent boundaries between LA regions. Figure 2b shows these 8 LA regions with the addition of the 16 PV quadrants (4 per vein). For validation, the defined LA-SUM regions were projected back onto the average LA shell (Fig. 2c). Examples of LA-SUM representations created from 3D LA maps representing LAT and LGE are shown on the left and right of Fig. 2d, respectively.

Representative LA-SUM maps from set A and set B are shown in Fig. 2e, f, respectively. In Fig. 2e, LA-SUM maps displaying LAT are shown for two cases during CS pacing and HRA pacing. In contrast to 3D LA shells, the LA-SUM displays activation times for the entire chamber in a single view allowing propagation of activation to be seen. Inter-observer differences between LA-SUM LAT maps were negligible (CS-RMSD = 0.007 ± 0.005 ms; HRA-RMSD = 0.008 ± 0.010 ms), confirming that the LA-SUM technique is robust to minor changes in the semi-automatic mesh pre-processing steps. In Fig. 2f, correlation between post-ablation CMR LGE and ablation FTI is shown for two cases in set B (one case per row). On the left of Fig. 2f, the LGE LA-SUM is shown, with black contours representing the identified regions of enhancement. On the right, these contours are overlaid in white on the FTI LA-SUM. Inter-observer differences between CMR-derived SUM maps were also minimal (FTI-RMSD = 0.068 ± 0.063 gs; LGE-RMSD = 0.031 ± 0.026 au).

**Fig. 3:**
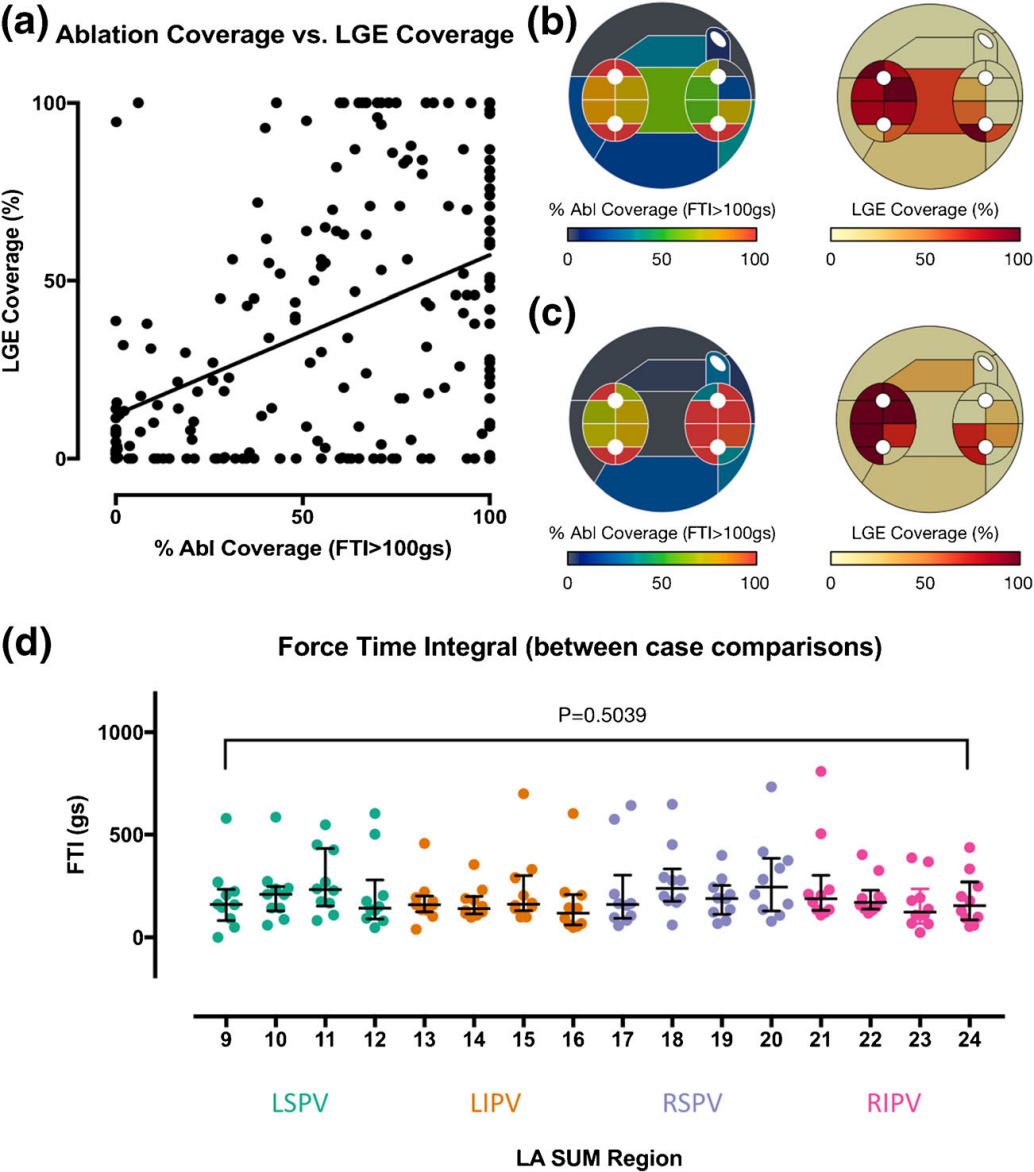
Quantitative intra- and inter-patient comparisons using the LA-SUM. **a–c** Intra-patient comparisons. Percentage coverage of LA-SUM regions during ablation (FTI > 100 gs) is compared with lesion formation assessed by LGE on post-ablation CMR imaging, showing a weak but significant correlation (**a**). Examples of cases showing close correlation (**b**) and weak correlation (**c**) between FTI and LGE on a region-by-region basis are shown. **d** LA-SUM facilitates inter-patient comparisons, demonstrated here for average FTI applied in each WACA quadrant for each vein during ablation

The use of the LA-SUM for intra-patient comparisons is demonstrated in Fig. 3a–c. Using the LA-SUM to quantify region coverage by ablation and by enhancement on post-ablation CMR revealed a weak but significant correlation between FTI coverage and LGE coverage (*R* ^2^ = 0.18, *P* < 0.0001, Fig. 3a). Examples of cases showing close correlation and weak correlation between FTI and LGE coverage on a region-by-region basis are shown in Fig. 3b, c, respectively. The use of LA-SUM for inter-patient comparisons is demonstrated in Fig. 3d, where the median FTI applied to each PV quadrant across all the cases in set B is represented. Overall, there was no significant difference between the median FTI applied at any of the quadrants (*P* = 0.5039) between any of the patients.

## Discussion

This study developed, validated, and demonstrated the application of a standardized 2D left atrial unfold mapping technique (LA-SUM). The rationale behind developing the LA-SUM representation was to provide a reference template facilitating the comparison of different datasets, both within patients and between individuals. Such unfolded visualization has long been used in the ventricle owing to several advantages: (1) it allows intra-patient comparison of multimodal data in a single view; (2) it provides a common reference space for inter-subject analysis; (3) data visualization does not require user interaction (i.e., map manipulation); and (4) unfold maps can be easily included in clinical reports.

The experiments on set A demonstrate the consistency of the LA-SUM technique. By manually defining LA regions on each 3D LA surface, prior to LA-SUM processing, the final LA-SUM representation of all cases in set A displayed the position of each region without bias from the original geometry. As can be seen from Fig. 2a, the final position of each region on the SUM map was highly consistent between cases. Given the variation in LA morphology seen in patients with atrial fibrillation [2], this robustness provides a unique method of comparing data between LA subregions of different cases. Consistent with their use for PVI procedures, the 3D LA shells in set A were characterized by detailed PV anatomy and sometimes a protrusion marking the position of the trans-septal puncture. Despite these differences in mesh anatomy, LA-SUM representations could be successfully computed for all cases.

Compared to traditional 3D LAT maps, LA-SUM LAT maps have the advantage of allowing the observer to visualize the activation of the entire chamber without requiring any user interaction as demonstrated for two cases under HRA pacing and CS pacing in Fig. 2e. Such LAT representation may be particularly useful for the visualization of atrial tachycardia circuits, where the entire mapped cycle length could be visualized on the single disk of the SUM map. The advent of ultra-high resolution mapping technologies leading to extremely detailed atrial maps will be ideally suited to this application [13].

Datasets from set B were used to demonstrate the use of the LA-SUM technique for performing intra- and inter-patient comparisons. For example, overlying multimodal information onto the LA-SUM facilitated comparison of LGE CMR images with ablation contact force on the same coordinate system. The data presented here show a weak but significant relationship between FTI and LGE which is consistent with prior working indicating that factors other than force (e.g., ablation power, catheter stability) are as important in the success of lesion generation [14]. Although thresholding was used in the present study to overlay datasets (e.g., white lines in Fig. 2f), we have previously developed a tool (termed dot mapping) for representing multiple scalar datasets in a single spatial domain [5]. Combining dot mapping with the LA-SUM would allow simultaneous visualization of multiple pan-atrial arrhythmia substrate characterization modalities.

Numerous previous studies have performed regional analysis of structural and electrical parameters in the LA [15–19], and yet to date there is no standardized method of comparing regions between patients. The LA-SUM provides a tool for performing such inter-patient comparisons. For example, in Fig. 3d, the LA-SUM was used to compare the average FTI applied in each PV region between ten cases undergoing PVI. By providing a reproducible method for LA regional assessment, the LA-SUM could enable standardization of future studies performing arrhythmia substrate characterization of the left atrium.

## Limitations

The proposed LA-SUM template was designed for the most common topological LA variant with four pulmonary veins (74% [20]). Modification of the template would be required to support LA-SUM generation of five-vein or common ostia morphologies.

## Conclusions

This study developed an approach to compute a template standardized unfold map of the left atrium. This approach is applicable to multiple types of input data and displays a unified holistic unfold for multimodal information. It also allows computing metrics per region in an automatic manner. The proposed standardized unfold map for the left atrium is analogous to the bull’s eye plot for the left ventricle. To facilitate use of this technique, code for generating the LA-SUM will be made available in an online repository.

## Electronic supplementary material

ESM 1 (DOCX 110 kb).
