# Supplementary material for: Standardized unfold mapping: a technique to permit left atrial regional data display and analysis
Source: J Interv Card Electrophysiol. 2017 Sep 7;50(1):125–31. doi: 10.1007/s10840-017-0281-3 (PMC5633640; doi:10.1007/s10840-017-0281-3)
Supplement: Supplementary file 1 — (DOCX 110 kb). [file 10840_2017_281_MOESM1_ESM.docx]

# Semi-automatic segmentation of the LA blood pool

The LA blood pool masks were extracted using a previously developed segmentation algorithm, implemented in NiftyTools ^1,2^. 19 atlases from the left atrial segmentation challenge^3^ were propagated to each patient’s WH dataset and fused to obtain a final segmentation ^4^. The mask was manually corrected when necessary using ITK-SNAP ^5^. The mask was used as input for the LA scar segmentation algorithm, which outputs a mesh with LGE values obtained from the image ^6^.

# Mesh standardisation and registration

Mesh standardisation was performed as follows. First the surface is remeshed using Poisson reconstruction ^7^ to remove irregularities and/or aliasing artefacts. To impose the same topology on the surfaces, the mitral plane and the PVs 10mm distal to the ostia were clipped with custom-built software. For the WH derived meshes, the mitral valve annulus position was computed automatically. For the electroanatomical meshes, the original mitral valve annulus was retained. The PV clipping required manual selection of four seed points (one per main PV).

Mesh registration was performed as follows. Each patient’s surface was registered to the average mesh using an affine transformation matching the PVs centroids and the mitral valve annulus. Subsequently, elastic registration of the meshes was performed using surface matching via currents ^8^.

# References

1. Zuluaga MA, Cardoso MJ, Modat M, Ourselin S. Multi-atlas propagation whole heart segmentation from MRI and CTA using a local normalised correlation coefficient criterion. *Lect Notes Comput Sci (including Subser Lect Notes Artif Intell Lect Notes Bioinformatics)* 2013;**7945**:174–81.

2. NIFTK - A Translational Imaging Platform. http://cmictig.cs.ucl.ac.uk/research/software/software-nifty (21 April 2017)

3. Tobon-Gomez C, Geers AJ. Left Atrial Segmentation Challenge 2013. https://github.com/catactg/lasc (21 April 2017)

4. Tobon-Gomez C, Geers AJ, Peters J, Weese J, Pinto K, Karim R, *et al.* Benchmark for Algorithms Segmenting the Left Atrium From 3D CT and MRI Datasets. *IEEE Trans Med Imaging* 2015;**34**:1460–73.

5. Yushkevich P a., Piven J, Hazlett HC, Smith RG, Ho S, Gee JC, *et al.* User-guided 3D active contour segmentation of anatomical structures: Significantly improved efficiency and reliability. *Neuroimage* 2006;**31**:1116–28.

6. Knowles BR, Caulfield D, Cooklin M, Rinaldi CA, Gill J, Bostock J, *et al.* 3-D visualization of acute RF ablation lesions using MRI for the simultaneous determination of the patterns of necrosis and edema. *IEEE Trans Biomed Eng* 2010;**57**:1467–75.

7. Kazhdan M, Bolitho M, Hoppe H. Poisson Surface Reconstruction. *Proc Symp Geom Process* 2006;61–70.

8. Durrleman S, Prastawa M, Charon N, Korenberg JR, Joshi S, Gerig G, *et al.* Morphometry of anatomical shape complexes with dense deformations and sparse parameters. *Neuroimage* Elsevier Inc.; 2014;**101**:35–49.
